# Adverse Events Following Measles-Mumps-Rubella-Varicella Vaccination and the Case of Seizures: A Post Marketing Active Surveillance in Puglia Italian Region, 2017–2018

**DOI:** 10.3390/vaccines7040140

**Published:** 2019-10-07

**Authors:** Pasquale Stefanizzi, Paolo Stella, Domenica Ancona, Katia Nicoletta Malcangi, Francesco Paolo Bianchi, Sara De Nitto, Davide Ferorelli, Cinzia Annatea Germinario, Silvio Tafuri

**Affiliations:** 1 Department of Biomedical Science and Human Oncology, Aldo Moro University of Bari, 70124 Bari, Italy; malcangikatia@gmail.com (K.N.M.); frapabi@gmail.com (F.P.B.); sara.denitto.ch@gmail.com (S.D.N.); cinziaannatea.germinario@uniba.it (C.A.G.); 2 Apulian Regional Health Department, The Strategic Regional Agency for Health and Social Affair of Puglia (AReSS Puglia), 70126 Bari, Italy; p.stella@regione.puglia.it (P.S.); domenica.ancona@auslbatuno.it (D.A.); 3 Interdisciplinary Department of Medicine, Aldo Moro University of Bari, 70124 Bari, Italy; davideferorelli@gmail.com

**Keywords:** safety vaccine, vaccine confidence, pharmacovigilance, febrile seizures, reporting rate, causality assessment

## Abstract

Since 2012, the Italian Ministry of Health has recommended to improve the surveillance of adverse events following the measles-mumps-rubella-varicella (MMRV) tetravalent vaccine that was provided in the official immunization schedule of some Italian regions for children during the second year of life. This recommendation was based on data from some surveys that showed an additional risk of seizure following the administration of this vaccine. Responding to the Ministry commitment, the Puglia Region launched, from May 2017 to November 2018, a post-marketing active surveillance program of adverse events following MMRV immunization (AEFIs). Immunized children (second year of life) were enrolled on a voluntary basis, AEFIs diaries were used, and their parents were interviewed 25 days after the immunization. There were 2540 children enrolled; 2149/2540 (84.6%) completed the post-vaccination follow-up. Of these, 992 AEFIs were registered with a reporting rate of 46.2 × 100 doses: 883/992 (89.0%) AEFIs were not serious, while 109/992 (11.0%) were serious. For serious AEFIs, the evaluation of causality assessment was performed using the algorithm proposed by the World Health Organisation (WHO): 82/109 consistent causal associations to MMRV immunization were detected (reporting rate of consistent AEFIs: 3.8 × 100 follow-up). All serious AEFIs consistently associated with immunization resulted completely resolved at the follow-up. The reporting rate of seizure consistently associated with immunization was 0.05 × 100, lower than data previous published in the literature that did not report the causality assessment. Because no emerging signals were detected, our data from the active surveillance program confirmed the safety profile of the MMRV vaccine.

## 1. Introduction

Vaccination is one of the greatest success and cost-effective intervention methods of modern public health. The benefits of vaccination in saving lives and promoting health through reducing the spread of infectious diseases has been recognized worldwide. No drug, medical procedure or immunization can be described as totally risk free. A vaccine is a very particular type of drug because it is administered to a great number of people, in major part in good health, including infants and children, so there is a low tolerance for potential risks of adverse events, even if vaccines are held to a higher standard of control than other medical products [1,2,3,4,5].

Adverse events following immunization (AEFIs) are medical occurrences following immunization, and they do not necessarily have a causal relationship with the vaccine: the majority are local and have short duration (not serious AEFIs) while serious AEFIs are absolutely rare [6,7].

The perceived risk of adverse events in the general public is the most important threat for implementing successful vaccination programs, because it may cause hesitancy, delays or refusal of vaccination. For this reason, pharmacovigilance activity of monitoring and assessing vaccine safety is a priority for public health [8].

In the post-marketing life of the vaccines, the World Health Organization (WHO) and National and International Drug Authorities (such as the Food and Drug Administration (FDA), or for Italy, the National Drug Authority (AIFA)) monitor the safety by collecting and analyzing reports of adverse events (passive surveillance) or by specific active surveillance programs [9].

Passive surveillance involves consumers (immunized people or their parents) and/or healthcare professionals who recognized and spontaneously submitted reports of AEFI to health authorities for subsequent investigation and response when required [10,11,12].

Although international guidelines recommended that all adverse events must be detected, passive post-marketing surveillance is affected by under-reporting, especially for non-serious adverse events, incomplete reporting information, biased reporting, with several difficulties to distinguish coincidental from causal events, and delayed notifications [13,14,15].

In 2006, Hazel et al. published a systematic review in which they estimate the extent of under-reporting of adverse drug reactions (ADRs) in spontaneous reporting systems, and the impact of under-reporting on public health decisions; They suggest possible initiatives to implement pharmacovigilance performance, such as specific active surveillance program improving, healthcare professional training, internet reporting, direct patient reporting and causal link evaluation [16,17].

According to Italian law, the notification of AEFIs is mandatory for health care workers, and patients can also report events potentially related to vaccinations to health authorities. Causality assessment evaluation for serious AEFIs is mandatory only since 2017, and the use of the WHO algorithm is recommended [7,18].

In September 2005, Food and Drug Administration and European licensed the quadrivalent measles-mumps-rubella-varicella (MMRV) vaccine for use among children aged 12 months to 12 years, and the Advisory Committee on Immunization Practices (ACIP) recommends a 2-dose vaccine schedule in childhood, with the first dose administered at age 12–15 months and the second dose at 4–6 years [19,20]. Some post-licensure studies about MMRV suggested a higher risk for febrile seizures 5–12 days after vaccination among children aged 12–23 months immunized by the MMRV vaccine, compared with children immunized by the measles-mumps-rubella (MMR) vaccine and the varicella vaccine administered as a separate injection at the same visit [21,22,23].

Febrile seizure is the most common neurologic, serious adverse event following immunization with measles-containing vaccines. Although febrile seizures are common in child under 5 years and not associated with long-term neurologic sequelae or developmental delay, they often frighten parents, which may precipitate acute care visits and undermine confidence in immunization programs [24,25].

Given the evidence for an increase in risk of febrile seizure, in 2009, ACIP adopted new recommendations regarding the use of the MMRV vaccine for the first and second doses, and identifying a personal or family (i.e., sibling or parent) history of seizure as a precaution for use of the MMRV vaccine for the first dose. ACIP also recommends that the real risk of febrile seizure and other AEFIs following the MMRV vaccine must be investigated intensively in post-marketing surveillance activities [26,27].

In last update fact sheet, the WHO reported that in 2018 the MMRV vaccine was adopted by five countries in Europe and two countries in America [28]. Since 2009, the MMRV vaccine has been marketed in Italy, and some regions (such as Apulia) recommended its use into Universal Vaccination Mass strategies for the elimination of measles and rubella and for the control of varicella [29].

In 2011, the Italian Drug Authority (AIFA) and Ministry of Health discouraged the continuation of the use of the MMRV vaccine, in particular for the first dose administered to children aged 12–23 months, in order to reduce the supposed increasing risk of febrile seizure; regions that choose to continue the use of the MMRV vaccine have to guarantee supplemental surveillance activities on the safety of the vaccine [30,31].

Puglia is a region in the south of Italy (4,000,000 inhabitants) in which the MMRV Universal Mass Vaccination started in 2009. The vaccine is offered actively and free of charge for children in the second year of life (first dose), and the second dose is administered at 5–6 years [22]. The vaccine is also used for catch-up strategies. Since 2017, vaccination against measles, mumps, rubella and varicella is mandatory in Italy [32]. The coverage achieved in the target cohorts exceeded 85% [33].

In order to answer to the commitment of the Ministry of Health, better monitor the safety of the MMRV vaccine and, at same time, improve the sensitivity and quality of the regional vaccinovigilance system, the Apulian Regional Health Authority planned and implemented an active surveillance program of adverse events following the first dose of MMRV immunization.

This paper summarizes the main results of this regional, post-marketing active surveillance program which lasted from May 2017 to November 2018.

## 2. Materials and Methods

The post-marketing active surveillance of adverse events following MMRV immunization was implemented from May 2017 to November 2018. The project was carried out in 12 vaccination clinics of the 6 Regional Districts.

The target population was represented by children for whom there is an active and free vaccination offer for the first dose of the measles-mumps-rubella-varicella vaccine (13–23 month of life), according to the Regional Immunization Schedule [31].

Immunized children were enrolled on voluntary bases and written consent was signed by one of the parents at the time of enrollment.

Exclusion criteria were:

a) Not the target age for active and free vaccination offered for the first dose of the MMRV vaccine;
b) Previous febrile seizures episode;
c) Contraindications (conditions in a recipient that increase the risk for a serious adverse reaction) and precautions [34].

Healthcare workers offered pre-vaccination counselling and explained the importance of AEFIs detection and the active surveillance program.

Parents received a post-vaccination diary in which they had to report all adverse events that occurred in the three weeks after vaccination (date of AEFIs onset, characteristics of the adverse events, case description, duration and treatment, hospitalization or emergency room access and final outcome).

Twenty-five days after the vaccine administration, a phone follow-up was carried out: parents of enrolled children were requested to report information of AEFIs noted in the post-vaccination diary. Data from medical records in the case of hospitalization were also collected.

If the AEFIs were not resolved at the time of the phone follow-up, a second follow-up was planned one month later.

All detected adverse events were inserted in National Pharmacovigilance Network.

WHO guidelines have been used to classify AEFIs as ‘serious’ or ‘not serious’ [7]. An AEFI is considered serious, if: it results in death; it is life-threatening; it requires in-patient hospitalization or prolongation of existing hospitalization; it results in persistent or significant disability/incapacity; it is a congenital anomaly/birth defect or requires intervention to prevent permanent impairment or damage. Additionally, in 2016, AIFA published a list of particular health conditions that, if happened after vaccination, must be considered as serious AEFIs [35].

For serious AEFIs we applied the WHO causality assessment algorithm to classify AEFI as ‘consistent causal association’, ‘inconsistent causal association’, ‘indeterminate’ or ‘not-classifiable’. The causality assessment was carried out separately by two public health physicians, experts in vaccinology; in case of disagreement, a third physician was consulted.

For every enrolled subject, a specific form was built, including information on date of birth, gender, date of the vaccine administration, vaccine eventually administered in the same visit (in the Apulian region the first dose of MMRV immunization is scheduled in the same visit of the first anti-hepatitis A vaccination) and AEFIs-related information (date of onset and date of computing in the National Pharmacovigilance Network, characteristics of the adverse events, case description, duration and treatment, hospitalization or emergency room access and final outcome); for serious AEFIs information of causality assessment evaluation was added.

Compiled forms were put in a database created by Excel spreadsheet and data analysis was performed by STATA MP14 software (Stata Corp LP, 4905 Lakeway Drive, College Station, Texas USA).

Continuous variables were described as mean ± standard deviation and interquartile (IQR) range, categorical variables as proportions, with the 95% confidence interval (95% CI), when appropriate.

AEFIs reporting rate was calculated using, in the numerator, the number of AEFIs reports, and in the denominator, the number of subjects enrolled in the study for which a follow-up was available.

Ethical statement:

All the investigations were carried out following the rules of the Declaration of Helsinki, Puglia. The research protocol must be submitted for consideration, comment, guidance and approval to the Puglia Regional Ethics Committee before the study begins.

## 3. Results

From May 2017 to November 2018, 2,540 children were enrolled. Twenty-five days after vaccine administration, the telephone follow-up was carried out for all the enrolled children, and it was available for 2149/2540 (response rate: 84.6%) subjects. Data about these subjects were discussed in this paper. The average age of enrollment was 14.8 ± 4.2 months. At follow-up, 992 AEFIs were detected (reporting rate: 46.2 × 100 enrolled children). The median time between vaccine administration and AEFIs onset resulted 5.0 days (range IQR = 1.0–8.0; range = 0.0–55.0), while the time between the onset of adverse events. In AEFIs forms, 91/992 (9.2%) reported the administration of the MMRV-vaccine, while 901/992 (90.8%) reported simultaneous administration of the MMRV and other vaccines: in 897/901 (99.6%) anti-hepatitis A (HAV), in 2/992 (0.2%) anti-meningococcal B vaccine and in 2/992 (0.2%) anti-diphtheria-tetanus-pertussis and polio vaccine (DTap-IPV) simultaneous administration.

Fever/hyperpyrexia is the symptom/clinical sign most frequently notified (*n* = 828/992; reporting rate = 38.5 × 100), but more than one symptom/clinical sign has been registered in tAEFI-reports (Table 1).

In AEFIs, 109/992 (11.0%) were classified as serious (reporting rate = 5.1 × 100), while 880/992 (88.7%) as non-serious (reporting rate = 40.9 × 100); 3/992 (0.3%) were not defined (reporting rate = 0.1 × 100). Of these, 8/109 (7.3%) must be considered serious AEFIs because children needed hospitalization, and 101/109 (92.7%) because particular health conditions (listed by AIFA in 2016) occurred [34].

The symptom/clinical sign most frequently notified in serious AEFI reports is fever/hyperpyrexia (*n* = 101/109; reporting rate = 4.7 × 100; Table 2).

Performing causality assessment, 82/109 (75.2%, reporting rate = 3.8 × 100) serious AEFIs were classified as ‘consistent causal association’ to the MMRV vaccination, while 1/109 (0.9%, reporting rate = 0.05 × 100) was indeterminate and 26/109 (23.9%, reporting rate = 1.2 × 100) were classified as ‘not consistent causal association’; no serious AEFIs was considered as not classifiable.

Fever/hyperpyrexia was detected in all ‘consistent causal association’ serious AEFIs (82/82, reporting rate = 3.8 × 100 follow-up) (Table 2).

At the time of first follow-up contact, 72/82 (87.8%) serious AEFIs classified as ‘consistent causal associated’ to the MMRV vaccine were already resolved. However, the other 10/82 (12.2%) serious AEFIs classified as ‘consistent causal associated’ were completely resolved at subsequent follow-ups.

## 4. Discussion

In our study, 2149/2540 children immunized with the MMRV vaccine were enrolled and analyzed: the pre-vaccination counselling of the importance of AEFIs detection probably favored adherence to protocol and correct diary updating. Response rate (84.6%) was consistent with literature data: in a recent study published by Regan A.K. et al., response rate to text messaging and oral voice telephone interviews for AEFIs detection ranged between 63.9% and 90.1%, depending on the age and sociodemographic characteristics of the enrolled people [36,37].

In our study, 992 AEFIs reports were registered, with a reporting rate of 46.2 × 100 doses administered. It is entirely expected that active surveillance data will show higher adverse event frequencies than passive surveillance systems, which are frequently characterized by multiple limitations, including unconfirmed diagnoses, under-reporting (especially for non-serious adverse events) and lack of clarity about the temporal link between AEFI and vaccination. A previous, similar observational study published by Huang W.T. et al., based on the telephone interview approach, estimated a very similar AEFIs reporting rate of 48 × 100 follow-up [38,39,40].

Fever/hyperpyrexia was the symptom/clinical sign most frequently detected (*n* = 828/992; reporting rate = 38.5 × 100): in general, incidence of adverse events resulted consistent with pre-licensure safety data [19]. Of the AEFIs, 109/992 (11.0%) were classified as serious (reporting rate = 5.1 × 100).

The proportion of non-serious adverse events resulted higher than the Italian estimate that indicated for 2017 in the last AIFA report (88.7% vs. 80.0% in AIFA report) and higher than Apulia date from passive surveillance in the 2013–2017 period (88.7% vs. 75.4%): this finding seems to indicate that the under-report of passive AEFIs surveillance mainly regarded non-serious adverse events [41,42].

We tested the use of the last updated algorithm of the WHO causality assessment to verify causal link association between vaccine and serious AEFIs to provide an adequate picture of vaccine safety. Of these, 82/109 (reporting rate = 3.8 × 100 follow-up) serious AEFIs resulted associated to vaccination and all regarded adverse events already known; therefore, no emerging signals were detected. Indeed, all consistent serious adverse events were completely resolved. In the previous studies about vaccine safety, the use of the causality assessment algorithm has not been implemented and it thus becomes difficult to compare the results [43].

Clonus/febrile seizures (the most common adverse event following the MMRV vaccine) were detected in 4/109 serious AEFIs reports (reporting rate = 0.2 × 100 follow-up). However, 2/4 clonus/febrile seizures were not consistently causal associated with immunization, because of the presence of alternative cause of adverse events; 1/4 were indeterminate because the time (8 days) from vaccination to adverse reaction onset is compatible, but another cause of hyperpyrexia and febrile seizure was supposed during hospitalization (viral pharyngotonsillitis); 1/4 (reporting rate = 0.05 × 100) were consistently consistent causal associated to MMRV vaccination. However, it was an episode reported by the mother of the child and characterized by clones during hyperpyrexia with dyskinesia and involuntary movements of the head, which was resolved spontaneously without hospitalization.

Data of our study were consistent to literature findings. In a recent study published by Cocchio et al. [44], fever was the most common systemic reaction after the MMRV vaccine, and the incidence of febrile seizure was 0.2%. Also, this study was based on active surveillance (then, the reporting rate was expected as higher) but causality assessment was not carried out, therefore we cannot evaluate the link between the vaccine and AEFIs [44,45].

In general, the use of the causality assessment seems to suggest that the real frequency of seizure consistently associated with the MMRV vaccine is lower than those published in the previous studies that considered all seizure temporary associated with the vaccination, without a standardized causal evaluation.

Previous observational study suggested that the MMRV vaccine is the only one for which biological plausibility (1–4 cases/100,000 doses) with the thrombocytopenia (petechiae, ecchymoses) has been confirmed in a time interval of 1–6 weeks after vaccination [46]. In our program of the MMRV active surveillance program, no thrombocytopenia adverse events related to the MMRV vaccination were registered for the number of subjects enrolled.

Post vaccination follow-ups demonstrated that all serious adverse events consistently associated with immunization were completely resolved. For this reason, although the number of AEFIs registered was higher, the active surveillance program confirmed and reinforced the safety profile of the vaccine. Data seemed to support that the MMRV vaccine is safe, and the choice of Apulia Region of continuing its use (despite the Italian Drug Authority and Ministry of Health recommendation in 2011) was right.

The main strength of our study probably can be considered the high quality of AEFI reports (no serious AEFIs report was not-classifiable) and the systematic use of the causality assessment; comparing with other studies, the rate of AEFIs is lower because we consider only serious AEFIs with a causal association with the vaccine [43].

In our study, the number of enrolled children is high, but it is not able to detect very rare adverse events (such as thrombocytopenia). For this reason, multi-centric studies can be implemented in order to improve the study sample and to analyze the feasibility of the causality assessment approach on a large scale.

## 5. Conclusions

In conclusion, post-marketing active surveillance programs can be considered an effective solution to a real question: the public concerns about risks associated with immunization. Both health care providers and the general public should be educated about the necessity of AEFIs detection and health providers should be encouraged to report AEFIs, to better know the safety profile of vaccines, and to improve public confidence in immunization programs [47]. Moreover, spontaneous reporting systems (SRSs) are pivotal for signal detection, especially for rare events with a high drug (or vaccine)-attributable component, but active surveillance programs periodically have to be implemented in order to improve the overall performance of the pharmacovigilance system and validate data and emerging signals detected by spontaneous reporting activity [48,49].

## Figures and Tables

**Table 1:** Number and reporting rate of symptoms/clinical signs most frequently notified. Apulia Region, post-marketing active surveillance following measles-mumps-rubella-varicella (MMRV) immunization, 2017–2018.

Symptom/Clinical Sign	Number of AEFIs *	Reporting Rate (×100 Enrolled)
Fever, hyperpyrexia	828	38.5
Neurological symptoms Agitation, nervousness Sleep disorders Fatigue, weakness Seizure, clonus	605 508 49 42 6	28.2 23.6 2.3 2.0 0.3
Redness, skin rash, swelling, local pain	418	19.5
Gastrointestinal diseases	141	6.6
Excessive, inconsolable crying	39	1.8
Lymphadenitis	28	1.3
Allergic reactions	8	0.4
Other local signs/symptoms	164	7.6

* AEFIs—adverse events following immunization.

**Table 2:** Number and reporting rate of symptoms/clinical signs most frequently notified in serious adverse events following immunizations (AEFIs) reports and in serious AEFIs consistently causal associated with immunization. Apulia Region, post-marketing active surveillance following the MMRV immunization, 2017–2018.

Symptom/Clinical Sign	Number of Serious AEFIs * Reporting Rate (×100 Enrolled)	Number of Consistent Serious AEFIs Reporting rate (×100 Enrolled)
Fever, hyperpyrexia	101 (4.7)	82 (3.8)
Neurological symptoms Agitation, nervousness Fatigue, weakness Seizure, clonus Sleep disorders	63 (2.9) 50 (2.3) 7 (0.3) 4 (0.2) 2 (0.1)	44 (2.0) 38 (1.7) 4 (0.2) 1 (0.05) 1 (0.05)
Gastrointestinal diseases	38 (1.8)	33 (1.5)
Redness, skin rash, swelling, local pain	36 (1.7)	28 (1.3)
Lymphadenitis	19 (0.9)	16 (0.7)
Excessive, inconsolable crying	3 (0.1)	3 (0.1)
Allergic reactions	1 (0.05)	0
Other local signs/symptoms	46 (2.1)	25 (1.2)

* AEFIs—adverse events following immunization.
